# Understanding privacy concerns in ChatGPT: A data-driven approach with LDA topic modeling

**DOI:** 10.1016/j.heliyon.2024.e39087

**Published:** 2024-10-09

**Authors:** Shahad Alkamli, Reham Alabduljabbar

**Affiliations:** Information Technology Department, College of Computer and Information Sciences, King Saud University, Riyadh, 11362, Saudi Arabia

**Keywords:** ChatGPT, Privacy concerns, Generative AI, Twitter, Survey, Latent dirichlet allocation (LDA), Data categorization, Unauthorized access, Data exploitation, Personal input

## Abstract

This study investigates privacy concerns associated with ChatGPT, a prominent generative AI model, through a data-driven approach combining Twitter data analysis and a user survey. Leveraging Latent Dirichlet Allocation (LDA) topic modeling and data categorization techniques, the research identifies key areas of concern: 1) Privacy Leakage Due to Public Data Exploitation, 2) Privacy Leakage Due to Personal Input Exploitation, and 3) Privacy Leakage Due to Unauthorized Access. Twitter data analysis of over 500k tweets, supplemented by a survey of 67 ChatGPT users, reveals nuanced user perceptions and experiences regarding privacy risks. A Python program was used to improve a dataset of 500k tweets referencing “ChatGPT” during the data preparation stage. To get a refined collection of terms, steps included converting text to lowercase, eliminating mentions and hyperlinks, tokenizing, eliminating stopwords, and keyword matching to extract tweets about ChatGPT's privacy features. Once preprocessing was completed, there were 11k refined tweets. Results highlight significant apprehensions, particularly regarding unauthorized access, underscoring the importance of robust privacy measures in AI systems. The study contributes to understanding user concerns, informing policy decisions, and guiding future research on privacy in generative AI. These studies might improve ChatGPT and other AI systems' security and privacy. The public, corporations, researchers, lawmakers, and AI developers may all benefit from the useful information it provides in better understanding and managing privacy threats.

## 1 Introduction

**Fig. 1:**
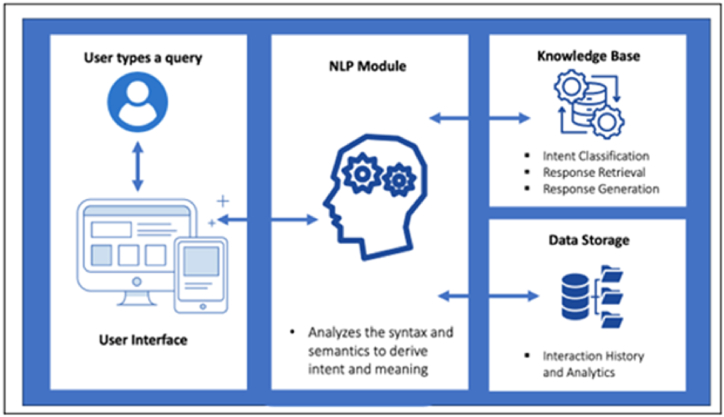
How Chatbot systems work? (David M., 2023 June 2)^39^.

Generative AI models are a prime example of the vast potential of unsupervised machine learning [1]. These models can produce various types of content, including text, audio, visuals, videos, and 3D models. Their profound impact extends to all aspects of our lives. One of the most promising developments in Generative AI is ChatGPT, a natural language processing system that generates human-like conversations. ChatGPT belongs to the category of Generative Pre-trained Transformers (GPT), which are language models [2]. ChatGPT is an advanced AI chatbot technology that uses NLP and machine learning to facilitate natural conversations between users and virtual assistants. Its design aims to achieve exceptional intelligence and intuition, allowing it to comprehend and respond to requests like a human [3]. Fig. 1 presents the main components and workflow of a chatbot system.

Generative AI and ChatGPT have a lot of potential, but it's important to consider the privacy concerns they raise. While ChatGPT can be useful in industries like business, education, healthcare, and content creation, it also raises questions about ethical boundaries and user privacy [[4], [5], [6], [7]]. Addressing these challenges is important for improving ChatGPT's effectiveness, usability, and overall user satisfaction, and for enhancing its performance across a wide range of applications and industries [8].

The implementation of built-in controls has helped to address concerns regarding individuals engaging in malicious activities like creating computer malware or password-cracking software. User privacy fundamentally involves protecting personal information considered confidential by individuals [7]. Generative AI tools such as ChatGPT can pose threats to user privacy in several ways. During the development of ChatGPT, extensive amounts of personal and private data are harnessed for training, posing a significant challenge in protecting individuals' privacy [9]. Concerns also extend to the potential exposure of private information, whether through deliberate actions or inadvertent breaches [4]. These are just some of the multifaceted risks that merit consideration.

### 1.1 Privacy concerns, the gaps, and the significance: the privacy issues with generative AI models, such as ChatGPT, are varied and fall into many main categories

i) Users must be concerned that ChatGPT and other AI models could utilize their public data, like postings on social media or public records, without getting their express permission. This might result in the creation of material that uninvitedly discloses private or sensitive personal information.
ii) Users face the risk of their private input, such as private messages or sensitive data, being stored or misused by the AI model. This raises concerns about data security and protecting sensitive information.
iii) Consumers could worry that unauthorized access to their interactions with AI models might be possible due to criminal activity or security holes in the system. Unauthorized access may lead to misuse or exposure of private conversations or data.

The gaps in the existing literature demonstrate the need for more thorough study and preventive strategies to successfully address the above issues. The specifics and complexities of user privacy concerns in generative AI remain largely unknown, even though research in the field provides useful knowledge. Due to several problems, it is essential to address the significance of privacy issues.

1) To build and preserve user trust in ChatGPT and other AI systems, privacy concerns must be addressed. If privacy safeguards aren't sufficient, users may not want to engage with these technologies, which would limit their adoption and potential benefits.
2) Consent from users and data security must be in line with ethical and legal requirements for user privacy. Businesses using AI models may be subject to regulatory scrutiny and legal repercussions if privacy concerns are not effectively handled.
3) In the digital age, privacy protection is essential to preserving people's freedom and worth as fundamental human rights. AI systems must protect and uphold users' rights to privacy
4) A violation of privacy may result in fraud, financial loss, damage to one's reputation, and psychological suffering, among other types of harm. By reducing privacy risks in AI models, we may lessen these negative effects and advance user welfare.

In conclusion, building trust, respecting moral and legal obligations, protecting individual rights, and avoiding user damage all depend on understanding, resolving, and minimising privacy risks in ChatGPT and other generative AI models.

With ChatGPT's growing integration into our society, it becomes increasingly vital to gain an understanding of the associated privacy risks and take measures to mitigate them [10]. In order to ensure that the users of ChatGPT have a positive and secure experience, it is of utmost importance to have a thorough understanding of their perceptions regarding the security and privacy concerns associated with ChatGPT. As such, this paper aims to explore the various discussions and conversations on social media platforms such as Twitter, to gather valuable information about the most prominent discourse on the topic. By doing so, we hope to identify the key areas of concern and take the necessary measures to address them to enhance the overall user experience of ChatGPT. Hence, the primary goal of this paper is to present an overview of existing privacy concerns of using LLMs such as ChatGPT as perceived by people on social media platforms. It gives information on the important areas where people are concerned and which require attention from authoritative bodies and the government. This information can help in shaping the policies regarding security and cyberattacks. For this, three steps were identified, firstly, we analyzed 11k tweets using keyword analysis, sentiment analysis, and LDA. Secondly, we conducted an online survey to gather insights from users about their privacy concerns. Lastly, we categorized Twitter data into identified privacy concern categories using keyword-driven classification methods. The contributions of this study can be categorized into the following headings.

1. The study presents a data-driven approach for understanding the privacy concern of ChatGPT providing insights into the nature as well as extent of privacy-related concerns.
2. It provides the concerns related to privacy from the user's point of view, hence, shedding light on real-world experience-based findings.
3. The process of topic modelling is employed to identify key areas of privacy concern pertaining to instances where users perceive their privacy to have been compromised. These areas are then flagged for remediation measures to strengthen security protocols and restore user confidence in the privacy framework.
4. The study's findings provide valuable insights into the privacy concerns of chatbot systems. By addressing these issues and incorporating relevant features, we can enhance the security and reliability of chatbots, making them more user-friendly and trustworthy.

The rest of the paper is organized as follows. Section 2 highlights the previous research on Generative AI and ChatGPT ethics and user privacy. In Section 3, the methodology employed in the study is delineated. The subsequent Section 4 presents the results and discussion of the study. Lastly, Section 5 concludes the paper by summarizing key findings and suggesting potential future research directions.

## 2 Related works

ChatGPT is a Large-language-based model (LLM) which have been now extensively used in almost every field including medicine, social sciences, education and even clinical applications. However, major concerns regarding its accountability, accuracy and security have been raised [36]. Since, its inception several authors have attempted to understand the implications, impact, strengths and limitations of ChatGPT. Alawida et al. [37] provid ed a detailed account of ChatGPT and identified several limitations such as biased predictions, misunderstanding the context of the cequery, absence of common sense, need for a large number of computational resources, requiring a large amount of data to perform a new task, and lack of interpretability.

The authors noted some of the ethical challenges in employing generative AI, including harmful or inappropriate mate-rial, prejudice, over-reliance, abuse, and the most critical obstacle, privacy and security. The papers emphasized the significance of resolving privacy and security concerns [[4], [9], [39], [40], [41], [42]]. The authors advised users to use caution while sharing sensitive personal or confidential information. AI businesses, particularly digital behemoths, should raise user understanding of ethical problems, such as limiting trade secret leaking, by offering guidance on what to share and what not to share with generative AI. Furthermore, the paper underlines the importance of strong legislation and policies to preserve information privacy and security. The authors of [38] presented a detailed overview of how ChatGPT can be used to commit and facilitate crimes using online platforms. They discussed how they can be used for hacking online systems in cases of jailbreaking. It also discussed the process of reverse psychology to manipulate responses from ChatGPT. They argued that through reverse psychology criminals can have a basic knowledge of fundamental workings and can manipulate inputs to take advantage of the AI's predictive capabilities, causing it to generate results that defy its morally-motivated design. This type of manipulation draws attention to a crucial component of AI weaknesses, which is the AI's vulnerability to inputs intended to subvert its anticipated reaction patterns.

The authors provided an analysis of the privacy concerns in ChatGPT. The paper used examples to highlight the possible hazards associated with using language generation models like ChatGPT [12]. One of the primary worries regarding ChatGPT revolves around its potential for generating sensitive or private data [13]. Another concern is that the model will be used to target individuals with tailored phishing, fraud, or other criminal actions. An attacker, for example, might utilize ChatGPT to construct a customized message that appears to have come from a bank or a person the victim trusts to get sensitive information or money from the victim [14]. To address these risks, the authors have suggested a set of technical precautions that can be implemented. These precautions include the use of techniques such as differential privacy, secure multiparty computation, federated learning, encryption, anomaly detection, access control, ongoing monitoring, and periodic evaluation. It should be noted that the efficiency of these techniques may vary based on the specific circumstances and data employed. The difficulties and dangers that GenAI may pose to privacy and security were examined by the authors in Ref. [15]. The study outlined ChatGPT's flaws, which might be used by malicious users to leak sensitive information. They brought up OpenAI's claim that it relies on “legitimate interests” when utilizing personal information about individuals as training data, which raises moral and legal concerns regarding how AI systems handle personal information, regardless of whether the data is made public or not. Also, they mentioned a data breach that happened in ChatGPT, this breach violated user privacy as it exposed their chats to third parties in an unexpected way. They suggested a solution to prevent sensitive information from being added to a ChatGPT's library, which is the ability to erase messages from the history of a ChatGPT or the simple fact that it doesn't save a user's conversation history.

The authors in Ref. [16] explored the data privacy and security concerns of ChatGPT and other AI tools. While utilizing ChatGPT for language learning, users may share personal ideas, feelings, and experiences. The collecting, storage, and use of such sensitive material may be risky. They asked several questions including: Are users properly informed about ChatGPT's methods for gathering data? Do they have any type of control over how their data is shared and used? Are there reliable measures in place to protect learner privacy and stop data misuse? Unfortunately, we have yet to obtain clear answers to these questions. In their conclusion, they highlighted the significance of placing privacy protection as a top priority to enhance the benefits of AI tools like ChatGPT while mitigating potential risks, ultimately leading to a more effective environment.

An analysis of the privacy concerns in using ChatGPT is provided by the authors in Ref. [17]. They found that many of the databases ChatGPT may access are derived from the Internet, including social media platforms like Twitter, implying that ChatGPT may use content that compromises personal information and lacks fact-checking. Therefore, it won't just provide inaccurate or misleading information, it will also compromise people's privacy. Following this, the authors provided a set of recommendations aimed at various stakeholders to ensure the responsible utilization of ChatGPT. They suggest that for researchers and developers, it is essential to take on the responsibility of educating individuals about privacy concerns and advocating for those who may not fully comprehend ChatGPT's disclaimers, particularly vulnerable members of society. Additionally, for users and consumers, the authors recommend a thorough understanding of the terms and conditions before initiating usage.

With a special focus on OpenAI's ChatGPT, the authors in Ref. [18] examined the crucial challenges of data security and privacy improvement in the context of LLM (Large Language Models)-based chatbots. They evaluated the Privacy-Enhancing Technologies (PETs) that were already in use and offered cutting-edge approaches including data minimization techniques, federated learning, and differential privacy. An online survey of chatbot users was also included in the study to figure out their worries about data privacy when using these LLM-based services. The majority of the survey participants expressed worries about privacy and data protection and agreed that AI systems should adhere to data protection laws. The survey showed that a sizable majority of users have prior experience with LLMs and have serious worries about their privacy and the safety of their personal information when utilizing these systems. There is broad agreement that AI systems must follow data protection laws. Participants emphasized that data anonymization, aggregation methods, and differential privacy should be used in AI systems. They also expressed concerns over possible security flaws like data poisoning and adversarial attacks, while demonstrating a readiness to give up some performance in exchange for better privacy and data protection. These results highlight the critical need for coordinated efforts to improve data security and privacy in AI systems.

The authors in Ref. [19] thoroughly examined the security risks associated with using ChatGPT and similar AI chatbots. They conducted a detailed survey as part of their investigation to better understand these risks and vulnerabilities. The survey revealed a significant finding: most respondents believed that ChatGPT could potentially collect personal information or manipulate users. Then, the authors listed various cyber concerns related to Chatbots like ChatGPT, including social engineering attacks, identity theft, and data leaks. All of these concerns have the potential to violate user privacy. To address these issues, the authors suggested some mitigation methods, including the implementation of strict laws to monitor and regulate the usage of ChatGPT. These laws should cover cybersecurity, data protection, and consumer rights, ensuring responsible and ethical use of the system while safeguarding the interests and rights of all users. With similar objective of exploring the awareness level among users, the study by Alawida et al. [34] presented a set of examples illustrating how ChatGPT can be a cybersecurity threat to us. The study participants included common users as well as IT experts and showed that while non-experts may not always be as aware of ChatGPT, computer experts were highly aware of it. As a result, continuing education and awareness-raising campaigns are required to address cybersecurity risks related to ChatGPT use.

In the realm of ChatGPT and its associated privacy concerns, existing research has left certain gaps. Previous studies have primarily relied on the analysis of ChatGPT privacy issues, primarily drawing upon prior research findings. However, our investigation revealed a limited number of studies that took a direct approach by conducting surveys among ChatGPT users to assess their concerns regarding data privacy and their experiences. While these surveys are valuable, they may not provide a detailed understanding of user concerns and experiences, suggesting the need for additional data collection methods. In light of these observations, this research endeavors to address these gaps by adopting a data-driven approach that combines surveys and Twitter data collection, in addition to identifying and analyzing prominent privacy concerns, thus contributing to a better understanding of ChatGPT's privacy dynamics. Table 1 provides an overview of key studies addressing privacy concerns in the context of generative AI and specifically ChatGPT. The summarized works shed light on data security and potential risks associated with language generation models.

**Table 1:** Summary of related works on privacy concerns in generative AI and ChatGPT.

Ref.	Year	Study	Summary
[4]	2023	The authors discussed privacy concerns and ethical challenges in employing generative AI, including harmful content, prejudice, over-reliance, abuse, and privacy/security concerns. They emphasized the need for strong legislation and policies to address privacy and security issues.	The study highlighted the importance of resolving privacy and security concerns in the use of generative AI. It also stressed the need for AI businesses to raise user awareness of ethical problems and the responsible handling of sensitive information.
[9]	2020	The authors devised a structured framework for AI ethics and explored ethical considerations in AI development, including data security and privacy.	The study found that contemporary AI research often prioritizes performance metrics over ethical considerations, and consumers tend to prioritize factors like price and quality.
[12]	2023	The authors analyzed privacy concerns in ChatGPT, highlighting potential hazards and risks associated with language generation models.	The study suggested technical precautions such as differential privacy, secure multiparty computation, and access control to address privacy and security risks in ChatGPT.
[15]	2023	The authors examined privacy and security challenges posed by GenAI, focusing on ChatGPT's potential for leaking sensitive information.	The study pointed out ChatGPT's data handling concerns and proposed solutions to prevent sensitive information from being added to its library, including the option to erase messages from its history.
[16]	2023	The authors explored the ethical dimensions of using ChatGPT in language learning, with a focus on data privacy and security.	The study emphasized the importance of prioritizing privacy protection while using AI tools like ChatGPT to enhance benefits and mitigate potential risks.
[17]	2023	The authors examined privacy concerns related to ChatGPT accessing Internet-derived databases.	The study provided recommendations for researchers, developers, users, and ethicists to ensure privacy concerns of ChatGPT.
[18]	2023	The authors evaluated Privacy-Enhancing Technologies (PETs) and conducted an online survey of chatbot users to understand their data privacy concerns.	The survey showed that users have serious concerns about privacy and data protection when using AI systems and emphasized the need for better privacy and data protection measures in AI.
[19]	2023	The authors surveyed to understand the security risks and vulnerabilities associated with using ChatGPT and similar AI chatbots.	The study found that respondents had concerns about data collection and manipulation by ChatGPT. It listed various cyber concerns and suggested mitigation methods, including the implementation of strict laws.

## 3 Materials and methods

The methodology used in this study involved several key components: data collection, data pre-processing, topic modeling, and data categorization. Data was collected from Twitter and an online survey to capture public discussions and targeted insights into user privacy concerns related to ChatGPT. During the pre-processing phase, tweets were converted to lowercase, hyperlinks and mentions were removed, and text was tokenized to focus on relevant content.

Topic modeling using Latent Dirichlet Allocation (LDA) was performed to identify major discussion themes within the tweets. This step provided a high-level overview of the topics being discussed but was not directly used for categorization.

**Fig. 2:**
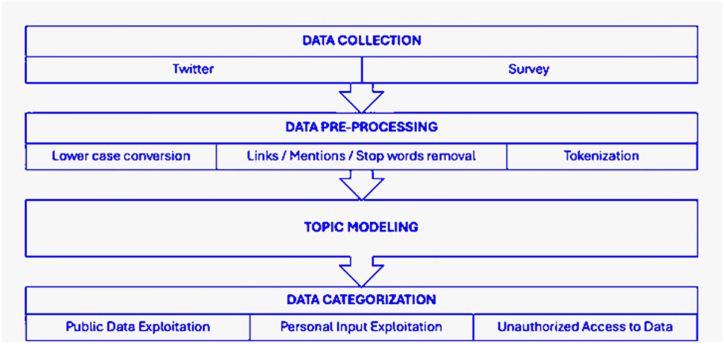
Methodology workflow.

For data categorization, tweets were systematically classified into three privacy-related themes: Public Data Exploitation, Personal Input Exploitation, and Unauthorized Access to Data. This categorization was based on keyword matching and was independent of the topic modeling process. This systematic approach facilitated a comprehensive analysis of the privacy concerns associated with ChatGPT, ensuring both broad thematic insights and detailed categorization of specific privacy issues. The methodology workflow is illustrated in Fig. 2.

Data Source

### 3.1 Twitter

The initial phase of data collection commenced with an attempt to access the Twitter API to extract relevant tweets for an in-depth analysis of ChatGPT-related discussions. However, due to Twitter's revised policy restricting access to only the most recent 7 days of tweets, this approach was inadequate for the complete temporal scope required for our research. To address this limitation, we shifted our focus to identifying an available dataset that could offer a broader view over time. The evaluation criteria considered were scale, temporal span, statistical properties, and user reviews. After a thorough assessment, a dataset meeting these criteria, sourced from the Kaggle website, was identified as the most suitable option.

The selected Kaggle dataset^1^ covers a substantial temporal span of three months immediately following the launch of ChatGPT on November 30, 2022. This timeframe was considered crucial for capturing the evolution of user attitudes and concerns during the early stages of ChatGPT's introduction. The dataset consists of over 500k tweets related to ChatGPT, providing a diverse range of discussions that reflect the dynamic nature of the ChatGPT community during this critical period. The dataset included a broad range of discussions about ChatGPT, we applied a set of additional keywords during the data preprocessing stage to focus on privacy-related tweets. These keywords included “security,” “privacy,” “cybersecurity,” “confidentiality,” “secure,” “hack,” “hacker,” “encryption,” and “theft.” By filtering the dataset with these keywords, we ensured that our analysis concentrated on privacy and security discussions within the context of ChatGPT.

One notable challenge during this phase was that the dataset was not specifically focused on privacy concerns related to ChatGPT but rather encompassed general discussions about ChatGPT. To address this issue, the next step, data preprocessing, played a pivotal role in refining the dataset. With thorough curation and filtering, we aimed to isolate tweets explicitly related to ChatGPT's privacy aspects. This strategic approach ensures that the dataset maintains its richness in diverse themes and user experiences while also becoming tailored to the specific incidences of privacy-related discussions within the ChatGPT community.

In summary, the sequential journey involved an initial attempt at API access, followed by the identification and selection of an appropriate dataset from the Kaggle website. It culminated in the recognition of the dataset's need for refinement to address the research focus on ChatGPT's privacy aspects. This process highlights the importance of adapting and strategizing in response to evolving constraints and research objectives.

### 3.2 Survey

In addition to the collection of Twitter data, a concise online survey^2^ was conducted to gain deeper insights into ChatGPT users' awareness, concerns, and experiences regarding privacy. The survey aimed to complement the analysis of Twitter data by providing a more targeted understanding of users' perspectives. The survey was administered through Google Forms over a two-week period in October 2023 and received a total of 67 responses from ChatGPT users.

To ensure a diverse sample, the survey was distributed through various channels, including social media platforms and local WhatsApp groups. However, it is worth noting that the majority of survey responses were obtained from users located in Saudi Arabia. While this concentration may introduce some geographical bias, it also offers unique insights into the privacy concerns of users from a specific region.

The survey included five key questions designed to explore different aspects of privacy concerns according to the categorization detailed in (section 3.4). These questions focused on concerns about inadvertently including private information in ChatGPT's responses, instances of users sharing personal information, worries about unintentional exposure of personal data in ChatGPT's responses, concerns regarding unauthorized data access, and experiences with privacy breaches.

To analyze the quantitative data obtained from the survey, descriptive statistics were employed. This analytical approach allowed for the summarization of the surveyed sample's characteristics and the identification of patterns in user privacy concerns related to the use of ChatGPT. By integrating this survey data into the research framework, the study gained a robust and empirical dimension, enhancing the ability to draw meaningful conclusions about the extent and nature of privacy issues in ChatGPT interactions.

### 3.3 Data preprocessing

In the data preprocessing phase, a Python code was used to refine the dataset of 500k tweets, all of which contained mentions of “ChatGPT.” The primary objective was to selectively extract tweets directly related to ChatGPT's privacy aspects. The preprocessing steps included.

1 Convert text to lowercase: This step ensures consistent analysis by converting all text to lowercase.
2 Remove hyperlinks: We used regular expressions to remove hyperlinks from the tweet text, as they do not contribute to the analysis of privacy aspects.
3 Remove mentions: Similarly, mentions (e.g., @username) were removed using regular expressions, as they are not relevant to the privacy analysis.
4 Tokenization: We used the NLTK library to tokenize the text into individual words. Tokenization breaks the text into words or phrases, which are then used for further analysis.
5 Remove stopwords: Stopwords are common words that do not carry much meaning (e.g., “and”, “the”, “is”). We used NLTK's list of English stopwords to remove these words from the tokenized text.
6 Keyword matching: A list of privacy and security-related keywords was curated, such as 'security', 'privacy', 'cybersecurity', 'confidentiality', 'secure', 'hack', 'hacker', 'encryption', and 'theft'. We applied keyword matching to filter and extract tweets that explicitly addressed ChatGPT's privacy dynamics.

For the pre-processing Pandas library [20] was used. To load the dataset into a DataFrame facilitating seamless data manipulation, and creating a structured foundation for operations. They are libraries [21], known for their regular expression capabilities, and played a crucial role in removing hyperlinks and mentions from the tweet text. Additionally, the NLTK library [22] was utilized for tokenization and stopwords removal, enhancing the depth of the preprocessing pipeline. The integration of these libraries significantly contributed to the clarity, efficiency, and organization of the data preprocessing pipeline.

### 3.4 Topic modeling

To gain a high-level overview and broader understanding of the major discussion topics and user concerns surrounding ChatGPT, we used Python along with LDA for topic modeling. LDA, or Latent Dirichlet Allocation, is a widely used algorithm in natural language processing that helps uncover latent topics within a collection of documents. LDA is a more practical solution for subject modelling than other methods due to its remarkable speed and efficiency.

The carefully curated set of processed tweets, encompassing a diverse spectrum of user interactions, played a pivotal role in constructing a complete document-term matrix through Python-based tools. We used the Gensim library [[11], [23]] in Python, which provides an implementation of LDA, to train the model on the preprocessed tweets. The LDA model discovers a set of topics based on the distribution of words in the dataset. Each topic represents a collection of words that frequently co-occur in the tweets. The model underwent rigorous training over 15 passes to ensure convergence and identification of privacy-related themes.

### 3.5 Data categorization

To further analyze the large dataset of tweets, a data categorization approach was undertaken to categorize the tweets and discern patterns and trends in thematic content. This data categorization process was conducted to systematically identify and understand the various dimensions of privacy risks associated with the use of ChatGPT, focusing on different aspects of data interaction and vulnerability. The categorization of privacy concerns was based on the following criteria.

• Source of Data: Identifying whether the data originates from public sources, user inputs, or internal system data.
• Nature of Risk: Determining the specific risks involved, such as unintentional data leakage, exploitation of user-provided information, or unauthorized access to system data.
• User Impact: Evaluating the potential consequences for users, including the exposure of sensitive information, breaches of confidentiality, and security vulnerabilities.
• Real-World Incidents: Considering documented cases and studies that highlight specific privacy breaches and their implications for AI systems and users.

Hence, through this process, our study identified three central categories.

1) Privacy Leakage Due to Public Data Exploitation: ChatGPT is trained on a vast corpus of publicly available text from the internet. Even though OpenAI takes precautions to remove sensitive and personally identifiable information (PII), it's challenging to ensure complete elimination [24,25]. If any private information remains in the training data, the model could inadvertently generate responses that contain this private information during interactions.
2) Privacy Leakage Due to Personal Input Exploitation: Users may share their data with ChatGPT, believing that their interactions are private and secure. For example, they may provide sensitive information like their full name, address, phone number, or other identifying details during conversations, assuming that the model will handle this data responsibly. However, there is a risk that this information could unintentionally appear in the model's responses or be inadvertently exposed to other parties, potentially compromising user privacy. Previously, a similar incident occurred when ChatGPT's chat history feature has been unavailable for an extended period because of a bug that inadvertently disclosed short descriptions of conversations from other users [26].
3) Privacy Leakage Due to Unauthorized Access: This can occur when malicious actors gain unauthorized access to the model's data, responses, or sensitive information [27]. Unauthorized access can take various forms, including data breaches, insecure API keys, man-in-the-middle attacks, session hijacking, insider threats, and even multi-step jailbreaking, as discussed in a recent research paper [[28], [29]]. Each of these poses a different set of risks to the privacy and security of the ChatGPT system and its users.

The data categorization process began with the utilization of keyword-driven classification techniques. The dataset, consisting of preprocessed tweets, was loaded from a CSV file into Python. Three dictionaries were initialized to organize the tweets into their respective categories. These dictionaries were created based on predefined sets of keywords associated with each thematic category, ensuring a distinct classification based on the semantic context of the tweet content. The keyword lists were generated with the assistance of ChatGPT (Table 2).

**Table 2:** Keywords used to retrieve relevant tweets.

Category	Keywords
Public Data Exploitation	public data, open data, sanitizing, training process, training data, trained on, publicly available data.
Personal Input Exploitation	conversation, conversations, input data, private chat, prompt, prompts.
Unauthorized Access to Data	unauthorized access, vulnerabilities, data breach, data security, security breach, attack, attacking, hack, hacking, threat, breach, unauthorized entry, data compromise.

The categorization function examined each tweet in the dataset and evaluated its content against the predefined sets of keywords. If a tweet contained a keyword associated with public data exploitation, personal input exploitation, or unauthorized access to data, it was categorized accordingly. This approach allowed for a granular categorization that reflected the diverse nature of the tweets within the dataset. After categorization, the tweets were organized into individual data frames for public data exploitation, personal input exploitation, and unauthorized access to data. These data frames were then merged into a unified dataset. To preserve the findings, the categorized tweets were saved to a CSV file for future analysis.

**Fig. 3:**
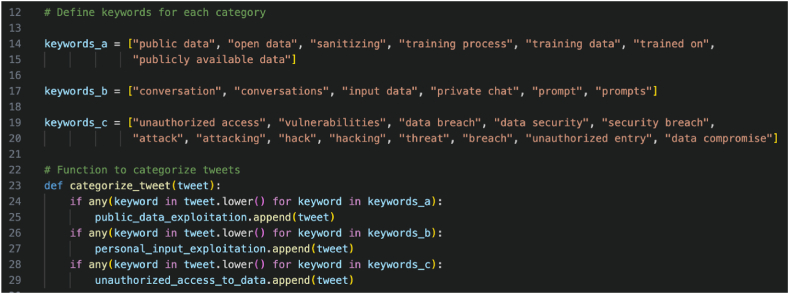
Implementation details of data categorization.

By employing a systematic data labeling approach, this research enabled the identification of patterns and trends in the thematic content of the tweets. This process facilitated a deeper understanding of the specific privacy concerns expressed by ChatGPT users, supporting the generation of insights and conclusions regarding privacy issues in ChatGPT interactions. The implementation details of the data labeling process can be found in Fig. 3, which provides a visual representation of the steps involved in segmenting and categorizing the tweets.

## 4 Results

### 4.1 Sample tweets after preprocessing

The data preprocessing produces a focused and refined dataset of 11k tweets, each intricately connected to ChatGPT's privacy aspects (Table 3). This dataset provided insights into the diverse perspectives, concerns, and considerations of ChatGPT users regarding the safeguarding of their data privacy within the context of ChatGPT interactions.

**Table 3:** Presents sample tweets after performing all the important preprocessing steps.

Unstructured Tweets	Structured Tweets (Preprocessed)
Ok. GREAT. I'm concerned about #ChatGPT privacy. Could we ∗∗really∗∗ enforce to forget data we gave ? https://t.co/iRHvqSlOd9	great concerned chatgpt privacy could really enforce forget data gave
Two #Cybersecurity Concerns When Using #ChatGPT For Software Development via @forbes https://t.co/m5tBWujmqwhttps://t.co/lTpqSVV86E	two cybersecurity concerns using chatgpt software development via
Sharing sensitive business data with ChatGPT and other AI Chatbots could be Risky! https://t.co/a1TNfNeHb6 #Cybersecurity #ChatGPT #AI #Chatbots #DataSecurity #Privacy #Security #BigData #TPRM #Modevity https://t.co/htno24WjB5	sharing sensitive business data chatgpt ai chatbots could risky cybersecurity chatgpt ai chatbots datasecurity privacy security bigdata tprm modevity
AI models like ChatGPT may lead to increased cybersecurity threats - Cyprus Mail - https://t.co/0vXDDnrzlf #GoogleAlerts @CyberNews #ChatGPT	ai models like chatgpt may lead increased cybersecurity threats cyprus mail googlealerts chatgpt
Security experts are warning that #hackers and #cybercriminals are misusing OpenAI's #ChatGPT, highlighting the need for quick regulation to address the potential risks of this technology. https://t.co/u89Nkf61QY #Phishing #Cybersecurity @ralexjimenez @junjudapi @jblefevre60	security experts warning hackers cybercriminals misusing openai chatgpt highlighting need quick regulation address potential risks technology phishing cybersecurity

### 4.2 Topic modeling

**Fig. 4:**
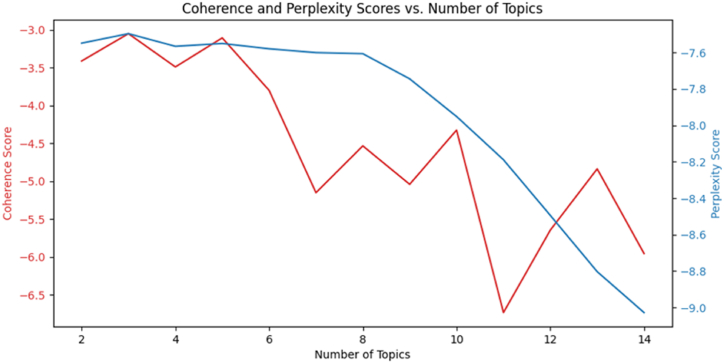
Coherence and Perplexity Scores vs. Number of Topics.

Based on the model evaluation metrics, including coherence and perplexity scores, we determined that the optimal number of topics for our dataset is 3. The coherence and perplexity score as a function of number of topics is plotted in Fig. 4.

**Fig. 5:**
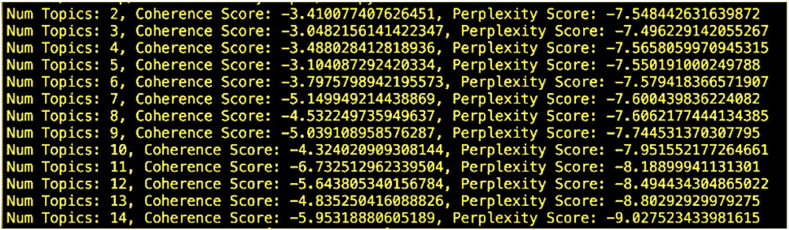
Coherence and perplexity scores.

**Fig. 6:**
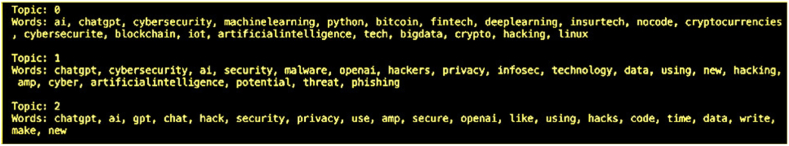
An overview of the topics and their corresponding keywords.

The coherence and perplexity scores are plotted in Fig. 5. An overview of the topics and their corresponding keywords is plotted in Fig. 6. Topics were then analyzed to identify specific privacy concerns expressed in the tweets, providing valuable insights into user perceptions and expectations regarding privacy when interacting with ChatGPT.

**Fig. 7:**
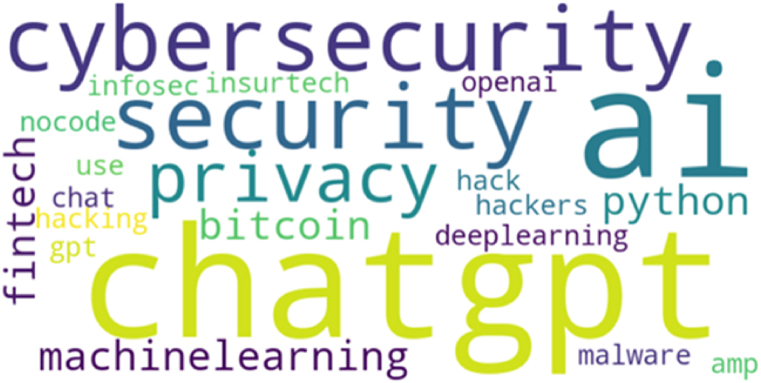
The most prominent keywords.

The next step involved extracting the most prominent keywords from the tweets. The visual representation of key terms is presented in Fig. 7, offering a succinct snapshot of the prevalent themes and focal points within the discussions.

### 4.3 ChatGPT's privacy concerns from Twitter

**Fig. 8:**
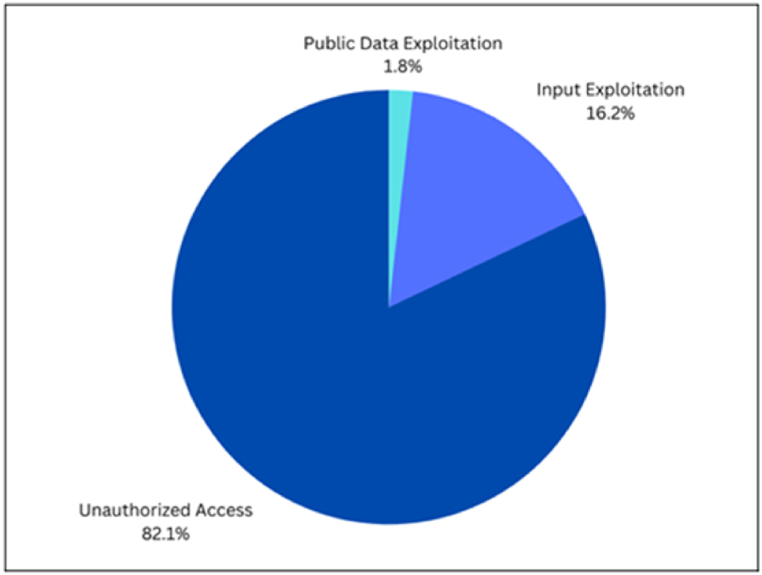
Twitter users' concerns about ChatGPT's privacy issues.

The analysis of Twitter data provides insights into the concerns of users regarding privacy issues associated with ChatGPT. This examination, based on a dataset of 11k tweets, categorizes expressions into three primary concerns: Privacy Leakage Due to Public Data Exploitation, Privacy Leakage Due to Personal Input Exploitation, and Privacy Leakage Due to Unauthorized Access. Fig. 8 presents a pie chart that visually represents the distribution of these concerns, providing a clear breakdown of the percentage for each category.

### 4.4 ChatGPT's privacy concerns from survey

To gain deeper insights into user awareness, concerns, and experiences regarding privacy, a survey was conducted with 67 ChatGPT users, building upon the insights from the Twitter data analysis.

#### 4.4.1 Privacy leakage due to public data exploitation

**Fig. 9:**
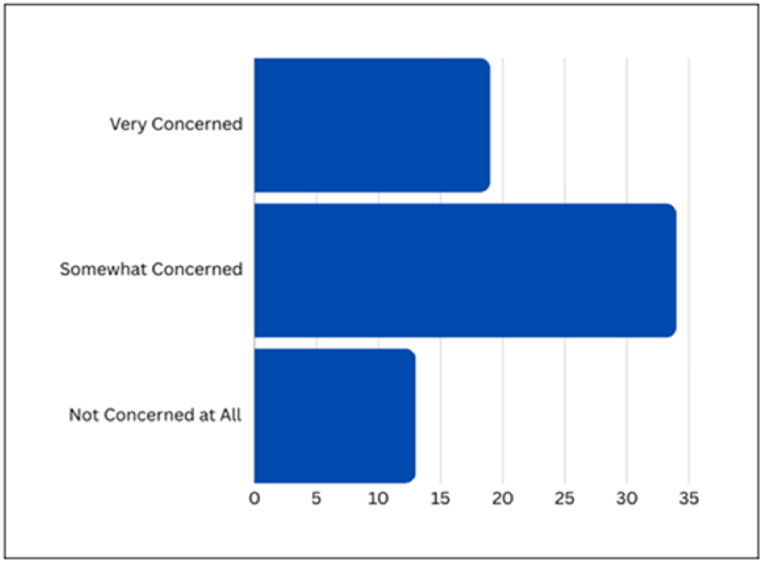
Survey responses on public data exploitation concerns.

Fig. 9 presents the survey outcomes concerning Privacy Leakage Due to Public Data Exploitation, providing a snapshot of respondent sentiments. The results indicate that 50.7 % of respondents express a 'Somewhat concerned' stance, 29.9 % are 'Very concerned', and 19.4 % assert they are 'Not concerned at all'.

#### 4.4.2 Privacy leakage due to personal input exploitation

**Fig. 10:**
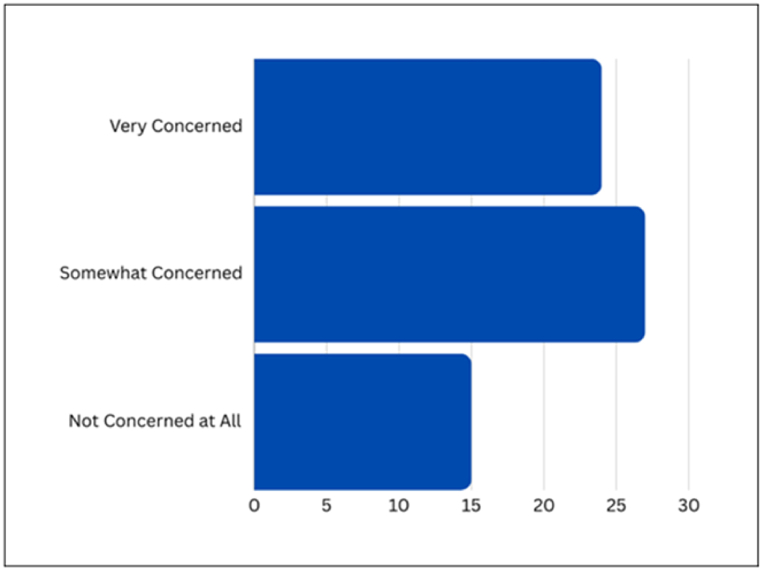
Survey responses on personal input exploitation concerns.

Fig. 10 presents respondent perspectives on Privacy Leakage Due to Personal Input Exploitation. The graphic representation shows that 40.3 % of respondents are 'Somewhat concerned', 37.7 % are 'Very concerned', and 22.4 % are 'Not concerned at all'.

#### 4.4.3 Privacy leakage due to unauthorized access

**Fig. 11:**
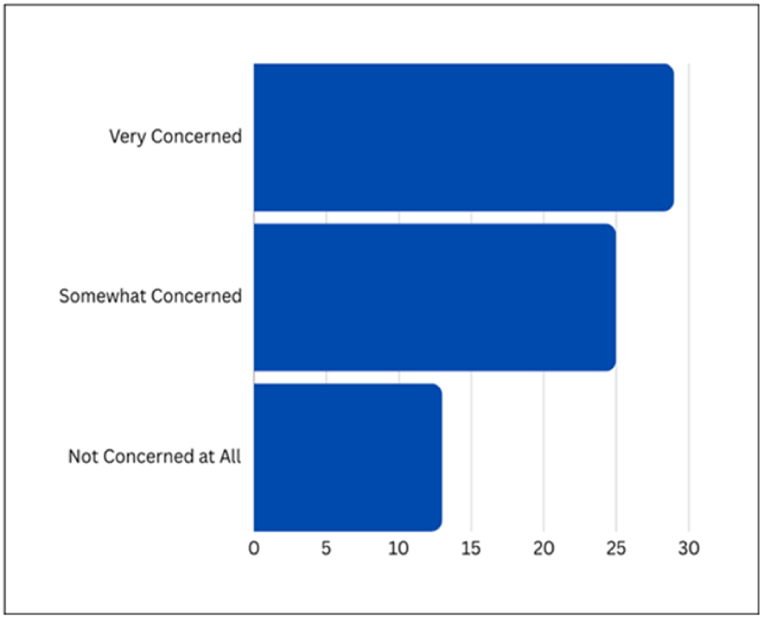
Survey responses on unauthorized access concerns.

In this section, Privacy Leakage Due to Unauthorized Access is explored, with Fig. 11 providing a visual breakdown of survey responses. The results show that a majority of respondents express concern, with 43.3 % being 'Very concerned', 37.3 % being 'Somewhat concerned', and 19.4 % asserting they are 'Not concerned at all'.

## 5 Discussion

### 5.1 Topic modeling

The topic modeling analysis conducted on the ChatGPT-driven Twitter dataset reveals three distinctive topics, each representing a different aspect of the discussions: In the first topic, users engage in discussions related to cybersecurity and emerging technology trends. They explore concepts such as blockchain, artificial intelligence, and big data, indicating a broader interest in the technological landscape. The presence of terms like “cybersecurity” suggests a concern for the security implications of these trends, including their impact on privacy. This topic reflects a proactive approach from users in understanding and discussing the broader implications of these technologies, possibly including how they affect the privacy features of ChatGPT.

The second topic directly addresses privacy concerns associated with ChatGPT. Users express worries about data security, privacy breaches, and potential misuse of AI technology. Terms like “privacy,” “security,” and “hackers” indicate a heightened awareness of the risks involved in interacting with ChatGPT. This topic highlights the need for robust privacy measures to protect user data and ensure secure interactions with AI-powered systems like ChatGPT.

The third topic delves into the practical applications of ChatGPT and AI technologies. Users discuss how to use ChatGPT securely and its potential vulnerabilities, indicating a mix of curiosity and caution. Terms like “hack,” “security,” and “data” suggest that users are interested in understanding the operational aspects of ChatGPT and its implications for privacy and security. This topic reflects users' desire to engage with AI technologies like ChatGPT while being mindful of the potential risks and challenges associated with them.

The topics identified through the topic modeling analysis align with the three categories of privacy concerns previously identified. These topics reflect users' awareness and discussions about cybersecurity, data security, privacy, and the potential risks associated with interacting with ChatGPT. While each topic may not directly correspond to a specific category, they collectively highlight users' overall concerns about privacy leakage due to public data exploitation, personal input exploitation, and unauthorized access. This alignment indicates that user discussions on Twitter are reflective of key privacy concerns associated with ChatGPT, providing valuable insights for understanding and addressing these concerns.

### 5.2 ChatGPT's privacy concerns from Twitter

According to the results obtain for ChatGPT's Privacy Concerns from Twitter, the majority of users on Twitter were primarily concerned about Privacy Leakage Due to Unauthorized Access, with 82.1 % expressing worries about unauthorized access to ChatGPT's data and responses. This indicates a significant level of concern regarding the security and protection of user data within the ChatGPT ecosystem. A notable portion of users, 16.2 %, also express concerns about Privacy Leakage Due to Personal Input Exploitation, reflecting apprehensions about sharing personal information with ChatGPT and the potential risks associated with it. However, only a small percentage, 1.8 %, express concerns about Privacy Leakage Due to Public Data Exploitation, suggesting that awareness of this particular issue may be limited among ChatGPT users.

These findings highlight the specific areas of concern within the ChatGPT user community, with a strong emphasis on unauthorized access and personal input exploitation. By understanding these concerns, developers and researchers can prioritize addressing the security and privacy aspects of ChatGPT, ensuring that user data is protected, and users feel confident in engaging with the system.

### 5.3 ChatGPT's privacy concerns from survey

#### 5.3.1 Privacy leakage due to public data exploitation

The analysis of data revealed several aspects of users’ fears. The substantial percentages under the 'Somewhat concerned' and 'Very concerned' categories indicated a collective worry, suggesting that users are mindful of the potential risks associated with public data exploitation. The presence of a cohort expressing no concern prompts further exploration into the factors influencing divergent attitudes. To effectively address these concerns, discussions and initiatives should aim to unpack specific aspects of public data utilization that contribute to user apprehension, fostering a tailored and user-centric approach to data management in AI systems.

#### 5.3.2 Privacy leakage due to personal input exploitation

Upon scrutinizing the visual representation, the survey data highlights a broad spectrum of user concerns, emphasizing a collective recognition of the imperative to safeguard personal input. The significant proportions within the 'Somewhat concerned' and 'Very concerned' categories underscore the cautious approach users adopt when revealing personal information. Additionally, when asked if they have ever shared personal information with ChatGPT during conversations, such as their full name, address, or phone number, 79 % of respondents responded negatively. This specific inquiry reinforces the overall trend of user wariness, indicating a prevailing reluctance among users to divulge sensitive information. This underscores the critical importance of robust privacy measures to address and alleviate user concerns surrounding data protection in AI interactions.

#### 5.3.3 Privacy leakage due to unauthorized access

Examining the visual representation further highlighted collective unease about unauthorized access, particularly evident in the high percentages under the 'Very concerned' and 'Somewhat concerned' categories. This underscores the critical need for robust security measures to protect user data and maintain trust. The visual representation serves as a catalyst for discussions on implementing security protocols, emphasizing the significance of user data protection in maintaining the integrity and reliability of AI systems.

The examination of ChatGPT users' privacy concerns, derived from both Twitter data and survey responses, provided an understanding of the diverse spectrum of user apprehensions. The prominent concern surrounding unauthorized access highlights the critical need for ongoing initiatives to address and mitigate privacy risks within AI models like ChatGPT. These insights significantly contribute to discussions on AI privacy and inform policy considerations. The dynamic nature of these concerns emphasizes the urgency for adaptive strategies in navigating the evolving privacy landscape inherent in generative AI models such as ChatGPT. Preserving user trust and data protection are paramount goals, making these findings integral to the development and deployment of AI technologies.

Analyzing these findings in the context of previous studies underscores the dynamic nature of user concerns. A thorough comparison reveals consistent patterns with existing research [[30], [31], [32]], particularly in the prominence of unauthorized access as a primary apprehension [[33], [34], [35]]. This alignment emphasizes the urgency for adaptable and responsive privacy measures within the AI landscape, building upon insights garnered from prior studies.

However, it is important to acknowledge the limitations of this study. The reliance on social media data, primarily sourced from Twitter, introduces inherent biases as the user base on these platforms may not fully represent the diverse range of ChatGPT users. Additionally, the survey's sample size is limited, which should be taken into account when interpreting the results. The study's focus on user perceptions intentionally omits a detailed exploration of the technical dimensions of ChatGPT's privacy measures, leaving room for future research to delve into these intricate technical aspects.

#### 5.3.4 Theoretical and practical applications

The present study on ChatGPT privacy problems offers significant theoretical insights as well as practical applications.

##### 5.3.4.1 Theoretical implications

• This study improves the theoretical understanding of privacy in the setting of generative AI models. The research improves previous privacy theories and models by identifying particular areas of concern, such as privacy leakage caused by public data exploitation, personal input exploitation, and unauthorized access. This deep knowledge fills gaps in existing privacy standards, which may not completely represent the complications provided by AI technology.
• Using Latent Dirichlet Allocation (LDA) to analyze privacy issues in a large dataset of tweets improves methodological approaches to privacy research. This method illustrates how topic modelling may be used to extract and categorize privacy-related problems, adding a new dimension to the study of user concerns in digital environments.

##### 5.3.4.2 Practical implications

• The results provide practical suggestions for enhancing privacy policies in AI systems like ChatGPT. Insights into user concerns about unauthorized access and other privacy problems may help developers design greater protection measures and privacy features.
• The findings are useful for policymakers and regulators developing guidelines for AI technology. The highlighted privacy problems may help to shape more focused policies and laws aimed at protecting user data and reducing the privacy risks connected with generative AI.
• By emphasizing common privacy problems, the research emphasizes the need for increased user knowledge and education. Organisations may utilize these findings to create more effective training materials and communication strategies that educate users about data management methods and privacy safeguards.

## 6 Conclusions

This study revealed the crucial need for a unified response from diverse stakeholders to address the prevailing privacy concerns surrounding ChatGPT. While the heightened awareness underscores an informed user base, it accentuates the critical importance of fostering and maintaining user trust. The evidence pointing to legitimate worries about privacy issues among users presents a dual awareness scenario that poses both a challenge and an opportunity. Effectively addressing these concerns becomes a pivotal challenge; however, it simultaneously provides an avenue to fortify trust through the implementation of robust privacy measures. As ChatGPT continues to integrate into various societal domains, establishing a trustful relationship between developers, policymakers, and users becomes paramount for the responsible and ethical deployment of AI technologies in our ever-evolving digital landscape. The collaborative efforts of these stakeholders will play a pivotal role in shaping a secure and ethical AI landscape globally, emphasizing the need for ongoing research, dialogue, and adaptive strategies to ensure the continuous improvement of user privacy in AI interactions.

## CRediT authorship contribution statement

**Shahad Alkamli:** Writing – review & editing, Writing – original draft, Visualization, Validation, Software, Investigation, Formal analysis, Data curation. **Reham Alabduljabbar:** Writing – review & editing, Writing – original draft, Validation, Supervision, Resources, Project administration, Funding acquisition, Conceptualization.

## Ethical approval and consent

Ethical approval was not required for this study because it did not involve the participation of human or animal subjects. Since the research focused on analyzing publicly available Twitter data and conducting an online survey that did not collect any identifiable information or involve any direct interaction with individuals, it did not meet the criteria for ethical review typically required for studies involving human or animal subjects. Therefore, the researchers determined that ethical approval was unnecessary for this particular study.

Informed consent was not obtained from participants in the online survey conducted for this study. Participation in the survey was voluntary, and respondents were not required to provide any identifying information. The survey questions were designed to explore users' perspectives on privacy concerns related to ChatGPT interactions, and all responses were collected anonymously. This approach was chosen to ensure participant privacy and encourage candid responses. Additionally, the survey introduction explicitly stated that participation implied consent for the use of responses in the research analysis.

## Data availability statement

The data presented in this study are available on request from the corresponding author.

## Funding

This research project was supported by Researchers Supporting Project number (RSPD2024R905), 10.13039/501100002383King Saud University, Riyadh, Saudi Arabia.

## Declaration of competing interest

The authors declare that they have no known competing financial interests or personal relationships that could have appeared to influence the work reported in this paper.

## Footnotes

1 Accessible at https://www.kaggle.com/datasets/khalidryder777/500k-chatgpt-tweets-jan-mar-2023.

2 Accessible at https://forms.gle/GVbUz9gUdarWsJQ29.
